# Cell and Gene Therapy in Equine Ocular Disease

**DOI:** 10.1111/vop.70151

**Published:** 2026-02-02

**Authors:** Kimberly A. S. Young, Lauren V. Schnabel, Brian C. Gilger

**Affiliations:** ^1^ Department of Clinical Sciences, College of Veterinary Medicine North Carolina State University Raleigh North Carolina USA

## Abstract

Equine ocular disease is common and often challenging to treat using traditional methods. This has led to the development of new therapies. Like human medicine, veterinary medicine is adopting cellular and gene therapy as innovative approaches. Equine ocular disease is a particularly promising area for these techniques. Notably, immune‐mediated diseases (such as immune‐mediated keratitis and equine recurrent uveitis), ulcerative keratitis, and infectious ocular diseases are of interest. Several ocular gene therapy products are approved for use in humans, and more are currently being researched in veterinary medicine. In veterinary practice, cell therapy mainly involves multipotent stromal cells or mesenchymal stem cells (MSCs), which are also widely studied in human medicine. This review aims to summarize the status of cell and gene therapy in equine ocular disease and provide background on the principles behind these treatments, as well as insights from human medicine. Although many in vitro studies and case series exist, a significant research gap remains. Despite growing clinical use, there are limited controlled in vivo studies assessing their safety, routes of administration, or effectiveness.

## 1 Introduction

This paper reviews the current state of the use of cellular and gene therapy in equine ocular disease, along with discussions of emerging therapeutic areas. While these treatment methods have important differences and operate through distinct mechanisms, gene and cell therapy are often grouped together as a category of advanced biologics. Sometimes called the third platform or pillar of drug discovery, after small molecules (the first) and antibodies and biologics (the second), the introduction of gene and cell therapy represents a paradigm shift in treatment approaches for many disease states [1, 2]. Gene and cell therapy each offer unique benefits, but can also be used in synchrony in ex vivo gene therapy, where the genetic manipulation of whole cells occurs before their therapeutic infusion [3, 4]. The indications for their use, route of administration, optimal combination therapy, and impact of simultaneously administered drugs on the efficacy of the novel therapeutic are yet to be described.

In veterinary medicine, research and clinical use of cell therapy have mainly focused on equine musculoskeletal diseases. However, other systemic conditions, including eye diseases, have become targets for these innovative treatments. Equine ocular diseases, whether infectious, immunologic, or traumatic, are common [5]. Currently, the primary treatment for these conditions involves long‐term therapy (and in the case of immune‐mediated diseases, often lifelong) that is difficult to administer, costly, and frequently ineffective [6, 7, 8, 9, 10]. Unsuccessful medical treatments can cause pain, necessitate surgical intervention, or lead to blindness. These issues highlight the need for new therapeutic options leading many practitioners to turn to cell and gene therapy to address these cases.

Despite the growing use of cell therapy in clinical settings, there is a notable lack of controlled studies. Case reports and series, combined with in vitro and translational experimental studies, have established a foundation of knowledge. Gene therapy lags slightly behind in implementation on the clinic floor, with no equine gene therapies currently available commercially. While promising treatments currently in clinical trials offer hope for an accessible, reliable, and curative monotherapy [11, 12, 13], those currently being evaluated in veterinary medicine largely aim to improve chronic acquired disease management rather than cure. Therefore, the available treatment options are often used in tandem with traditional therapeutics as adjunctive therapies. This adds unanswered questions of interactions with traditional therapeutics and optimal combined drug protocols. With the increasing application of gene therapy in human medicine and the introduction of the first veterinary gene therapy to the market, reviewing the current state of our knowledge is timely [14]. This paper aims to discuss current applications, evaluated studies, existing knowledge gaps, and relevant translational research that could point to potential therapies in both therapeutic classes.

## 2 Cell Therapy: Definitions and Current State in Veterinary Medicine

The term “cell therapy” refers to the treatment using any live, whole‐cell‐based therapy that aims to ameliorate or cure a disease state [15]. These cells can be used at various stages of differentiation and can be either autologous or allogeneic. The benefits that cell therapy provides over its molecular counterparts are that whole cells have an innate ability to interact with the rest of the body system in a bidirectional crosstalk mechanism, allowing the cells to sense and then respond to a changing environment [2]. While whole blood transfusion, packed red cells, and platelet transfusion are technically “cell therapy,” this term generally refers to a more targeted administration of cells for a specific disease state, such as immune cell, microbiota, and stem cell therapy [16]. In veterinary medicine, cell therapy most commonly refers to stem cell therapy, which is the focus of this review. A summary of the current published literature relating to equine ocular disease and mesenchymal stem cells (MSCs) is provided (Table 1).

**TABLE 1:** Summary of published work involving mesenchymal stromal cells in equine ocular disease or equine ocular in vitro models.

Experimental model	Disease of interest	Source of stem cell	Dose and route	Key takeaway	Citation
Case series	IMMK	Autologous bone marrow‐derived	15 million cells q3–4 weeks for 3–5 doses	Resolution in 3 out of 4 clinical cases refractory to prior medical management; enucleation in remaining case due to worsening disease	Davis et al., 2019
Case series	IMMK	Autologous muscle‐derived	2.5–5 million cells Subconjunctival, q4–6 weeks for 4–7 doses	Reduction in lesion score after first injection, four; 4/6 eyes achieved complete recovery	Narinx et al., 2023
Case report	IMMK, ERU, Ulcerative keratitis, Retinal Detachment	Autologous Blood‐derived	Ophthalmic artery, intralesional, and/or subconjunctival Dose undescribed	Cases refractory to medical management responded to stem cell management	Marfe et al., 2012
Ex vivo co‐culture with peripheral lymphocytes from horses with ERU	ERU	Allogeneic adipose‐derived	5:1 CD4^+^ to MSC ratio in 12 well tissue‐culture plates	Decrease in activated CD4^+^ T‐cells found in ERU horses	Saldinger et al., 2019
In vivo safety and migration study	ERU	Allogeneic adipose‐derived; pIC activated and eGFP/luciferase‐labeled	Subconjunctival 10 million q2 weeks for three doses	Well‐tolerated (no clinical difference from PBS control); MSC components identified in subconjunctival phagocytes post‐mortem	Cassano et al., 2023
Case report	Ulcerative Keratitis	Blood‐derived, donor status unknown	Jugular vein and transverse facial artery; topically 125 000/injection site, single treatment 500 000 cells/5 mL drop solution applied topically three times a day for 7 days	Resolution of ulcer with minimal scarring after previously being refractory to traditional therapy	Spaas et al., 2011
In vitro migration assay	Ulcerative Keratitis	Allogeneic bone marrow‐derived	250 000 MSCs/transwell in six well plate	Faster wound closure with both MSC secretome and MSC co‐culture (fastest)	Sherman et al., 2017
In vivo toxicity study	ERU	Allogeneic and autologous stem cells, source undescribed	25 or 50 million intravitreal	Severe toxicity, including lymphoplasmacytic uveitis, retinal detachment, cataract, and phthisis bulbi	Moore et al., 2013 *abstract only, not peer‐reviewed*

Stem cells are defined by their ability to proliferate, self‐renew, and differentiate into specialized cell types. Whether these cells are multipotent or pluripotent is determined by the source of the cells, with embryonic‐derived stem cells and induced pluripotent stem cells exhibiting pluripotency and the more commonly used mesenchymal stromal cells (now preferentially referred to as multipotent stromal cells, or MSCs [17]) exhibiting multipotency [18, 19]. MSCs are often preferred due to their ease of access, safety profile, and the absence of ethical concerns associated with embryonic‐derived stem cells [20]. While the plasticity of the pluripotent stem cells, both embryonic and induced pluripotent stem cells, carries the risk of tumorigenicity [21]. Induced pluripotent stem cells (iPSC) carry the highest risk of tumorigenesis, particularly fatal teratomas [22, 23]. This plasticity can be beneficial for applications where engraftment and differentiation into a target cell type is the end goal; however, engraftment and differentiation are also unreliable, and even differentiated iPSCs maintain oncogenic properties [21]. The lack of pluripotency, and instead having limited differentiation capacity, decreases this risk for MSCs [24]. For a cell to be considered an MSC, it must be adherent, self‐renewing, fibroblast‐like cells that differentiate into osteoblasts, adipocytes, and chondrocytes in vitro [25]. They can be isolated easily from adipose tissue, bone marrow, umbilical cord, or muscle, and can differentiate into ectodermal and endodermal tissues in addition to the four definitive mesoderm lineages [20].

MSCs have been extensively researched and clinically used in both human and veterinary medicine, with veterinary medicine particularly benefiting from using animals as a translational model [19, 26, 27, 28, 29, 30]. The vast majority of equine studies and clinical applications focus on orthopedic diseases [31]. However, the benefits seen in musculoskeletal disease are immune‐mediated and tissue repair, which are broadly applicable to multiple organ systems [31]. Although the use of stem cells in equine medicine has become increasingly commonplace, there remains a significant lack of controlled, in vivo studies and evidence‐based guidelines for their use outside of orthopedic disease. Instead, the literature is predominantly focused on in vitro and case studies. This is particularly problematic for guidance on clinical implementation due to the difficulty in predicting cell behavior in vivo [29].

### 2.1 Paracrine Function of MSCs

Historically, MSCs have been targeted for the therapeutic end goal of engrafting into target tissues for regeneration. However, the fate of viable MSCs in the tissue is often short‐lived, whereas their therapeutic effects are long‐lasting [18, 32, 33]. This is thought to be due to their paracrine‐like effect, where MSCs secrete cytokines and chemokines, along with other bioactive substances, that alter the local microenvironment, encourage angiogenesis, improve tissue organization, alter inflammation, and recruit cell regeneration [34, 35, 36, 37]. There are numerous known secreted factors, and discovery is ongoing [31, 34, 35]. While their ultimate influences (known as trophic effects) fall into multiple categories, most focus on inhibition of fibrosis, immunomodulation, inhibition of apoptosis, and promotion of tissue regeneration [37]. Most applicable to the equine eye are their immunomodulatory, wound healing, and anti‐fibrotic effects. While an in‐depth review of each of their pathways and ultimate influences is beyond the scope of this paper, a brief review of important secreted factors is provided.

Appropriate, organized wound healing and the inhibition of pathogenic fibrosis are tied to the anti‐inflammatory properties of MSCs, due to the role of chronic inflammation in the formation of scar tissue and inappropriate fibrosis [38]. This is primarily accomplished by matrix remodeling, TGF‐β‐mediated differentiation into myofibroblasts, decreased collagen secretion by fibroblasts, and inhibition of oxidative stress [33, 38, 39]. Equine MSCs specifically have been shown to secrete tenascin‐C and plasminogen activator inhibitor‐1, which promote fibroblast migration, decrease fibroblast adhesion, and reduce anisotropy of dermal fibroblast actin in a dermal wound healing model, all aiding in wound closure [40].

MSCs have been extensively documented to have effects on natural killer cells, dendritic cells, B lymphocytes, and T lymphocytes, which make them an ideal target for the treatment of autoimmunity [29]. Immunomodulatory properties of MSCs can be altered significantly based on the microenvironment of the cell [34]. Polarization of MSCs into MSC1 and MSC2 subtypes has been described, with MSC1 exhibiting a pro‐inflammatory phenotype and MSC2 displaying an immunosuppressive phenotype [41]. This is postulated to be mediated by toll‐like receptors (specifically TLR3 and TLR4) [41]. These phenotypes represent a shift in the ratios of secreted factors. T cell regulation and neutrophil and macrophage activation are regulated by IL‐6, IL‐8, HLA‐G (or in the case of equine patients, the functional homolog EQMHCB2 [42]), IL‐10, IFN‐γ, PGE‐2, NO, IDO, IL‐1RA, and TGF‐β predominantly [31, 33, 34, 35, 36]. This is predominantly mediated through the MSC inhibition of T‐cell proliferation and their subsequent secretion of IFN‐γ has been evaluated in the horse and found to be mediated largely through PGE2 in vitro [43]. Both B‐cell and T‐cell suppression are purported to be downregulated through halting the G0/G1 phase of the cells [44, 45, 46].

### 2.2 Immune Privilege

While stem cells were once considered immune‐privileged, this is not universally true and varies from cell to cell, depending on the state of activation or upregulation of MHC II [47]. This can affect their therapeutic capacity due to cell survival and alteration of secreted factors; however, the extent of their immune response and ultimate clinical impact remains unclear [48, 49, 50]. Therefore, the question of using autologous or allogeneic stem cells remains. Allogeneic cells could be cultured in advance and made available off‐the‐shelf, allowing for prompt administration to the patient at the time of injury. Another benefit is the choice of donor, as age, medication, and disease state have previously been shown to impact donor cells and alter their secreted factors [51, 52, 53]. Autologous cells avoid the immunogenicity concerns but do require 2–3 weeks of tissue culture expansion time prior to administration.

Repeated dosing has also raised concern for immune stimulation. This was evaluated in 10 horses receiving multiple doses of allogeneic injections of 25 million adipose‐derived or bone marrow‐derived MSCs administered intravenously. While no clinical impacts were observed, the horses receiving BM‐MSCs had increased CD 8+ T cells [54].

To overcome these concerns for antigenicity, MSC in vitro culture methodologies for use during isolation and expansion prior to clinical application (often called priming or licensing) are being investigated [41, 55, 56]. As examples, allogeneic MSCs that were TLR4‐primed (exhibiting a pro‐inflammatory MSC1 phenotype) altered T cell interactions and resulted in permissive activation in in vitro co‐culture, whereas allogeneic MSC2 cells did not [41]. Furthermore, TGF‐β2 treatment has been shown to decrease MHC class I and II surface expression [57, 58, 59]. Since the interior of the eye has low levels of MHC class I inherently, it is unknown what impact this immunogenicity has on ocular disease, particularly as the cornea and ocular surface likely have higher expression [60, 61]. This may leave open more allogeneic options for ocular applications without in vitro manipulation.

### 2.3 Route of Administration and “Homing”

Mesenchymal stem cells can be administered through various routes for equine ocular disease, but the most common method is subconjunctival injection. Intravitreal injection has previously been shown to cause significant adverse events in humans and in horses, often leading to blindness or retinal detachment [62, 63, 64]. In the abstract resulting from an in vivo equine study cited (Moore et al.), seven out of nine injected eyes developed adverse effects [64]. Despite the lack of other published trials, intravitreal injection is contraindicated in the horse due to the consistency of adverse effects across species and the severity of the adverse effects. Topical ocular administration has also demonstrated therapeutic potential in canine KCS, suggesting its potential use in equine disease as well; however, administration frequency and cost may be concerns [50, 65]. Though not yet used broadly in the clinical setting, this may be able to overcome in the future via the use of a nanofiber, thermo‐responsive polymer, amniotic‐derived, or tissue‐engineered scaffold, which have also been implicated in enhancing the paracrine secretion of MSCs in culture in preliminary research [66, 67, 68, 69, 70, 71].

Direct administration is not required to achieve therapeutic efficacy at the target site with MSCs due to their paracrine effects. However, some features of MSCs do require contact with target cells (termed juxtracrine effects) [72, 73]. In this case, and in the cases of cell engraftment, the stem cell's innate property of “homing” or migrating to a site of inflammation or injury via transmigration across the endothelium into the peripheral tissues is utilized [74, 75]. While MSCs derived from multiple tissues have similar migratory pathways, there are some source differences in migratory capacity [76]. In both bone marrow‐derived and adipose‐derived MSCs, stromal‐derived factor 1 (SDF‐1) and CXCR4 interactions partially regulate the migratory capability of MSCs [77, 78]. Secreted PGE2 has also been implicated in the migration of MSCs [79]. However, the complete mechanism is unclear and has not been fully investigated in equine MSCs. Despite the lack of species‐specific mechanistic processes, it is known that equine MSCs will migrate to regions of injury after regional and intravenous perfusion [80, 81]. Homing in ocular tissues has been evaluated in other species (murine), where labeled MSCs administered intravenously were found in allografted corneas [82, 83]. In equine ocular administration, the fate of those cells injected subconjunctivally has been investigated, but only phagocytosed remnants of cells were identified after 2 weeks in the conjunctiva [84].

## 3 Gene Therapy: Definitions and Current State in Veterinary Medicine

Gene therapy relies on the delivery of genetic material to target cells and tissues to correct or compensate for disorders [85]. This genetic material can either directly deliver a copy of a faulty gene in a genetic disease or can aim to encode cells to produce proteins that will supplement, counteract, or modulate disease. These mechanisms allow the treatment of both congenital and acquired diseases [85]. Multiple gene therapies have recently entered the human market, many of which were tested in animal models [86].

Because gene therapy involves the delivery of nucleic acids, it requires a drug delivery mechanism. This can either be accomplished via a viral vector or a non‐viral vector [87, 88]. Viral vectors leverage the innate ability of viruses to invade cells, specifically those that rely on the insertion of genetic material into the host genome [87, 89]. Frequently used viruses include adenoviruses, adeno‐associated viruses, lentiviruses, and retroviruses, which all have varying properties for degree of cellular integration, persistence, immunogenicity, tissue penetration capacity, and tropism, genetic carrying capacity, and off‐target effects that guide the selection of each as a viral vector for an intended therapy [89]. Selecting viruses with appropriate tissue tropism can be beneficial in ensuring that the target transgenes are effectively delivered to the target tissues. Viral vector tropism was investigated over 15 years ago, when gene delivery by AAV5 to the equine cornea was successfully established [90].

While non‐viral vectors do not have the innate ability for easy integration and often require more advanced delivery systems, they lack some of the concerns of immunogenicity, antigenicity, and alterations to the host genome that viral vectors bring [89, 91]. These can include, but are not limited to, plasmids, overexpression systems, circular or linear covalently closed vectors, or minivectors, each carrying its own benefits and risks [87, 89, 91, 92, 93]. While less investigated, these have been evaluated both in vitro and in vivo in the horse for both ocular and non‐ocular related conditions [94, 95, 96, 97, 98]. A summary of published literature available is provided (Table 2). Therapies can be further divided into in vivo therapy, where the alteration of the target genome occurs in the patient, and ex vivo *therapy*, where target cells are removed and then modified prior to administration [27]. Both have been evaluated in a research setting in the horse [99].

**TABLE 2:** Summary of published work evaluating gene therapy with equine ocular applications.

Experimental model	Gene therapy mechanism	Transduced gene	Key takeaway	Citation
In vivo, intravitreal injection in healthy equines	AAV8‐mediated	Equine IL10 (overexpression)	High doses caused ocular inflammation; low‐doses well‐tolerated; limited environmental shedding; no systemic toxicity	Young et al., 2024
In vivo, murine experimental autoimmune uveitis	AAV8‐mediated	Equine IL10 (overexpression)	Single injection inhibited EAU and was well‐tolerated	Crabtree et al., 2022
In vitro, equine corneal fibroblasts	Polyethylenimine (PEI) nanoparticles	Decorin (overexpression)	Decrease in α‐smooth muscle actin (decreased fibrosis)	Donnelly et al., 2013
In vitro, equine corneal fibroblasts	Plasmid	Smad2, 3, 4 (silencing); Smad7 (overexpression)	Silencing of Smad2, 3, or 4 and overexpression of Smad7 resulted in inhibition of fibroblast transdifferentiation	Marlo et al., 2018
In vitro, equine corneal fibroblasts	AAV5‐mediated	EGFP	AAV5 can successfully transduce equine corneal cells	Buss et al., 2010

Similar to cell therapy, the majority of gene therapy research in equine medicine has focused on orthopedic diseases; however, the eye is a unique target organ that is well‐suited for gene therapy [1]. Ocular relative immune‐privilege shields some of the concern for immune response to foreign antigens [100, 101]. It is also a small size that is easily accessible for direct delivery, eliminating the need to travel through significant non‐target tissues [102]. Cell and gene therapy are also conducive to bypassing the challenges encountered when using traditional therapeutics, which are caused by the physical ocular barriers that can prevent drugs from permeating to the site of action [102].

As previously described, numerous facets of regenerative medicine have focused on the benefits of biologically active substances, such as growth factors, cytokines, and chemokines. However, when administered in traditional biologics, they are rapidly degraded. That opens the door for the use of gene therapy to make host tissue create its own bioactive therapeutics rather than requiring repeated administration [103].

While gene therapy holds immense promise as a tool for managing difficult‐to‐treat diseases, it also poses inherent risks. These risks differ between vectors, routes of administration, and in vivo versus ex vivo therapy. One concern when using viral vectors is the development of immunity to the viruses used [27, 58]. These can be assessed prior to administration using serum neutralizing antibodies to evaluate exposure and antibody generation. Capsid engineering can be utilized to evade immune recognition and is a crucial aspect of viral engineering for gene therapy [104]. Choice of a lower immunogenic virus (or one that was less likely to have previously induced an immune response in the patient) is also important in vector design [105].

## 4 Ex Vivo Cell Modification—The Combination of Gene and Cell Therapy

As previously mentioned, gene and cell therapy can be used in combination. This combination could enhance therapeutic capacity [4]. Gene therapy performed on whole cells or tissues ex vivo can enable the use of modified cells that enhance their therapeutic capacity [106]. An example of this technique that is changing the face of cancer treatment is the rise of chimeric antigen receptor T (CAR‐T) cell therapies, where T cells are removed from the patient, engineered to specifically target their unique cancer, and then reintroduced into the body [107]. While CAR‐T cell therapy has not yet been implemented in equine research, the use of ex vivo gene therapy has been investigated by modulating MSCs [108, 109, 110]. MSCs have been previously shown to be readily modulated by viral vectors [111].

## 5 Cell and Gene Therapy in Immune‐Mediated Equine Ocular Disease

As powerful immunomodulatory cells, MSCs have significant therapeutic potential in immune‐mediated diseases. These conditions are often lifelong and are typically managed with symptom relief rather than a cure. Consequently, ongoing use of traditional treatments with short half‐lives creates significant gaps in progress toward developing new therapeutic options.

### 5.1 Immune‐Mediated Keratitis (IMMK)

Immune‐mediated keratitis, the most common non‐ulcerative keratitis in horses, is characterized by intermittent corneal opacity, cellular infiltration, epiphora, and ocular pain. Beyond the superficial cornea, the disease may affect the stroma and endothelium [112]. The etiopathology of IMMK is unknown. Diagnosis is based on clinical presentation, response to therapy (immunosuppressive administration), and is mainly T‐cell mediated infiltrate [113]. Uncontrolled immune dysregulation can cause tissue damage and worsening corneal opacity that may threaten vision [112, 113, 114]. In translational models, MSCs have been shown to improve corneal opacity, decrease inflammatory cytokine levels, and reduce neovascularization [68, 115].

In vivo case series are the only published literature evaluating cell therapy in equine IMMK [116]. Davis et al. evaluated allogeneic equine bone marrow‐derived MSCs (BM‐MSC) delivered to four horses via subconjunctival injection every 3–4 weeks for 1–5 injections (15 million cells). In patients who were previously refractory to standard medical management, 75% showed a positive response to therapy, characterized by improved vascularization and corneal clarity [115]. Another case series by Narinx et al. evaluated six otherwise healthy horses that suffered from chronic IMMK previously unresponsive to medical management. These horses received a lower dose (2.5–5 million cells) of autologous muscle‐derived stem cells in 4–7 doses every 4 weeks. Initially, all horses responded positively. 60% of the horses ultimately achieved their disease being designated “in control.” [116] Finally, a single horse was diagnosed with treatment‐refractory IMMK. A single dose of autologous blood‐derived MSCs was administered intra‐arterially via the ophthalmic artery and subconjunctivally [117]. In addition to the lack of controlled clinical trials, interpretation of these results collectively is further complicated by the heterogeneity of the study designs, including different routes, doses, sources of stem cells, frequency of administration, and route of administration.

### 5.2 Equine Recurrent Uveitis (ERU)

ERU is a painful, recurrent disease that impacts 2%–5% of horses. It requires frequent, lifelong treatment to manage the painful and vision‐threatening disease [6]. It is the most common cause of blindness in horses [118]. ERU is predominantly characterized by T cells (predominantly CD4+) [118, 119]. The disease is characterized by epitope spreading of autoantigens resulting in a relapsing/remitting phenotype, with each bout accruing lasting cellular damage, which presents as scarring, cloudiness, and ultimately, vision loss [6, 120, 121].

Because we know that MSCs are potent modulators of T cell phenotype and proliferation, ERU is a tempting target for therapy. An in vitro study supported this finding by showing that the percentage of activated CD4+ T‐cells isolated from equine peripheral blood was decreased when co‐incubated with allogeneic adipose‐derived MSCs, with decreased expression of CD25, CD62L, Foxp3, and IFNγ. When prostaglandin pathways and cell‐to‐cell contact were interrupted, the shift in T‐cell phenotype decreased, suggesting that these pathways are important in modulation [122]. Safety of subconjunctivally‐injected adipose‐derived allogeneic MSCs that were pre‐stimulated with TLR‐3 agonist polyinosinic, polycytidylic acid (pIC) was investigated and showed no change in ocular scores in healthy horses [84].

Multiple targets for transgenes have been identified as potential therapeutic targets for ERU, including equine IL‐10, which was successful in reducing or resolving (respectively) clinical signs in in vitro murine experimental autoimmune uveitis models after intravitreal injection using an AAV8 vector [123, 124]. IL‐10 plays a crucial role in maintaining the anti‐inflammatory and immunosuppressive properties of the eye [125]. Inflammation leading to barrier malfunction results in a compromise to that immune‐privileged status. Transduction of IL‐10 through an AAV8‐eqIL‐10 intravitreal injection was moved from the murine model into an in vivo equine safety study in five healthy horses. Toxicity and shedding, as well as adverse clinical signs, were monitored for 3 months at escalating doses. Viral shedding was not detected in feces or urine, and viral genomes were not detected after day 3 in the tears of the horses that were injected. The injections were well tolerated with mild, transient, and self‐resolving inflammation. However, horses in the high‐dose group developed keratic precipitates on day 70 and 77. The inflammation was hypothesized to be caused by prolonged transgene expression allowed by IL‐10‐mediated viral immune escape [126].

## 6 Cell and Gene Therapy in Equine Ulcerative Keratitis

Equine ulcerative keratitis is the most common ocular disease in horses [5]. Blood‐based biologics, like platelet‐rich plasma, serum, and platelet‐rich fibrin, are already regularly used in the clinic [10].

A study in vitro used a scratch wound migration model to evaluate the healing rate of isolated equine stromal fibroblasts with equine allogeneic BM‐MSC, the supernatant or conditioned growth media of the BM‐MSC, or a control [127]. While both the BM‐MSC and the supernatant healed faster than the control, the BM‐MSC healed significantly faster. The concentration of transforming growth factor (TGF)‐β1 in the culture media was also increased in the BM‐MSC group compared to the control group. This increase in TGF‐β1 is consistent with murine studies showing similar elevations and is associated with fibroblast proliferation, increased integrin expression, and accelerated wound repair [127, 128]. Conversely, in vitro evaluation of the impact of TGF‐β1 on equine keratocytes and epithelial cells revealed inhibition of both cell lines at multiple doses [129]. TGF‐β1 also plays a role in myofibroblast differentiation, which influences corneal opacity and scarring. While it promotes extracellular matrix deposition, too much can promote uneven deposition [95].

In vivo case reports have found similar increases in wound healing. Both reports used peripheral blood‐derived MSCs administered systemically and topically. In one report, a case of bacterial keratitis, previously unresponsive to traditional medical management, resolved with minimal scarring after a single dose of autologous peripheral blood‐derived MSCs, administered both intravenously and intra‐arterially to the ophthalmic artery. MSCs were also applied topically three times a day for 7 days [130].

The other case report included four horses with keratitis of unknown infectious status that did not respond to traditional medical management. These horses were treated with one or two injections of IV and intra‐arterial (ophthalmic artery) administration of blood‐derived stem cells, along with topical therapy at various intervals. All reported resolution [117].

While gene therapy has not been evaluated for corneal healing in equine in vivo studies, methods to enhance wound healing and preserve corneal clarity have been evaluated in vitro in equine corneal fibroblasts. Donnelly et al. investigated a non‐viral polyethyleneimine (PEI) nanoparticle‐mediated gene transfer encoding Decorin (a proteoglycan and TGF‐β antagonist known to mediate collagen fibrillogenesis) to improve TGF‐β‐mediated corneal fibrosis in equine corneal fibroblasts. The PEI nanoparticles successfully transduced the cells without impacting viability and attenuated the transformation of fibroblasts to myofibroblasts [95]. Another study investigating the use of a non‐viral vector (plasmid‐mediated gene silencing) altered the expression of Smad genes in equine primary corneal fibroblasts [131]. Specifically, Smad2, 3, and 4 (all profibrotic) were silenced, while Smad7 (anti‐fibrotic) was overexpressed. Both profibrotic (Smad2, 3, or 4) gene silencing and antifibrotic (Smad7) gene overexpression ameliorated TGF‐β1‐induced myofibroblast differentiation [131].

## 7 Cell and Gene Therapy in Infectious Ocular Disease

Although no studies have directly examined the role of MSCs in infectious keratitis, equine MSCs are known to have antimicrobial properties that could be important in the eye. In an in vitro study, equine peripheral blood‐derived MSCs were found to secrete cysteine proteases [132]. These proteases have been shown to interfere with biofilms formed by MRSA, restoring antimicrobial effectiveness. The conditioned media, or supernatant containing the secreted factors of the MSCs, were applied to bacterial culture and found to reduce biofilm in other pathogenic strains relevant to bacterial keratitis, including Pseudomonas spp. [8, 132] Yet another study found that MSCs derived from equine peripheral blood inhibited the growth of both *Staphylococcus aureus* and *Escherichia coli* through paracrine activity, specifically identifying four secreted antimicrobial peptides (cystatin C, elafin, lipocalin 2, and beta defensin 2). When these were suppressed, their antimicrobial effects were mitigated [133]. Equine MSCs have also been shown to secrete CCL2, which stimulates the innate AMP expression activity of equine keratinocytes [134].

## 8 Other Uses of Gene and Cell Therapy in the Equine Eye

### 8.1 Retinal Detachment

A single case report of bilateral retinal detachment was treated with systemic steroids and topical antimicrobials for 2 months prior to administration of a systemic (IV) injection of peripheral blood‐derived MSCs [117]. Improvement in vision was noted 3 months after injection, with unilateral ultrasonographic improvement of the detachment. Though stem cells were used in treatment successfully, causality of the improvement cannot be established.

## 9 Focused Potential Future Applications in Equine Ocular Disease

### 9.1 Corneal Transplantation

While no studies have evaluated the use of MSCs in equine corneal transplantation, their use in human medicine in preventing graft rejection has been evaluated. Corneal transplantation from frozen donor corneas is generally well‐tolerated [135, 136]. However, vascularization that occurs in equine corneal transplants qualifies them all as having some degree of graft rejection [135]. In murine studies, there are data to support improved allograft survival with injection, though results vary based on timing of infusion and medications administered in parallel [137, 138, 139, 140]. In another murine study, intravenously injected MSCs were found to home to transplanted cornea successfully and improved allograft survival via inhibition of antigen‐presenting cell maturation and T‐cell suppression [82]. Alternatively, there are other studies showing that there is no improvement in allograft survival, reflecting the need for more controlled studies to evaluate route and timing of administration, source of the MSCs, and cellular dose [141, 142]. In an example of gene and cell therapy used in synchrony, murine bone‐marrow‐derived MSCs that overexpressed IL‐10 were generated via lentiviral transduction and doubled allograft survival time after subconjunctival injection compared to IL‐10 protein or BM‐MSC treated animals [143]. Therefore, controlled studies are needed to evaluate the potential for use of MSCs in equine corneal transplantation.

Multiple approaches for graft survival and improved clinical outcomes have been investigated using gene therapy in animal models [144, 145, 146, 147]. Both systemic and local therapy on the donor corneal tissue can result in effective transduction of target genes. In one in vivo murine study by Gilger et al., gene therapy was used to improve allograft survival in a corneal burn model. The transgene encoding a single‐chain immunomodulator (scIM), a chimeric immunomodulatory protein that mimics the activity of HLA‐G, was introduced via an AAV8 vector into the corneal allografts ex vivo. This resulted in improved corneal fibrosis and reduced corneal vascularization. When introduced into a high‐risk corneal transplant rabbit model, all of the non‐treated allografts failed; 83% of those treated survived [106]. Other cytokines and proteins of interest include IL10 [148], indoleamine 2,3‐dioxygenase (IDO) [149], p35 [150], the p40 subunit of IL12 in sheep [151], and programmed death‐ligand 1 (PD1) [152]. However, this list is not exhaustive and many other targets are being investigated due to the complex process of allograft rejection and, subsequently, the many steps to target to prevent it.

### 9.2 Corneal Regeneration

While the discussion in this review has largely focused on the paracrine benefits of MSCs, there are some data that MSCs can differentiate into corneal epithelium and (to a lesser extent) corneal stromal cells [153, 154, 155, 156, 157]. While these studies have been performed in MSCs from multiple sources, the ability to differentiate into keratocytes is most supported in corneal stromal stem cells (CSSCs) [156], which have been shown to be more similar to bone marrow‐derived MSCs than limbal epithelial stem cells in their gene expression patterns [158, 159]. Both bone marrow‐derived and adipose‐derived MSCs were shown to not only survive in the corneal stroma and increase keratocyte density, but also to exhibit some functional keratocyte activities after injection into the anterior chamber of rats [157]. Ultimately, a systematic review of published data from 2015 identified that 10 of 11 studies contained positive evidence of corneal cell marker expression by MSCs [160]. The meaning of this conclusion is somewhat questionable due to limited distinct markers for keratocytes [161]. Ultimately, there is also evidence that even in cases where differentiation did not occur, the inhibition of inflammation and angiogenesis encourage corneal reconstruction [162].

### 9.3 Postoperative Corneal Wound Healing

Postoperative scarring and corneal opacity have been ameliorated by MSC administration in non‐equine species [163]. Improved clarity and inflammation in healing corneal wounds is postulated to be due to secretion of TSG‐6 in response to injured corneal cells [164]. This improvement in transparency has also been replicated in numerous small animal models [165, 166]. Between the increase in corneal transparency and other secreted factors encouraging wound healing, tissue organization, angiogenic regulation, post‐operative application for corneal healing beyond transplantation is a potential application of MSCs.

Transduction of cells to promote corneal wound healing has been extensively investigated [144, 167, 168]. Genes of interest encode or silence proteins involved predominantly in vascularization, scarring, or fibroblast proliferation. The TGFβ signaling pathway is known to cause corneal scarring postoperatively, in which Smad proteins play an important role [169, 170]. In an in vivo rabbit model, an AAV5‐Smad7 gene therapy administered topically post photorefractive keratectomy reduced corneal haze and corneal fibrosis after a single application [171]. Beyond Decorin, which is discussed in the equine sections, other targets include matrix metalloproteinases (MMP 14) [172], modifications to the rapamycin (mTOR) signaling pathway [173], BMP7 [174], and HGF^174^.

## 10 Regulatory Mechanisms

Gene and cell therapy both have international and national regulatory challenges for study and for implementation in the sport horse. Gene therapy can be performed on germline cells or on somatic cells. Currently, there are no investigations into germline cell modification for clinical use due to concerns for the unintended consequences of future generations acquiring modified genes in horses [87, 175]. However, some argue that changes to germline cells may be worth the risk of off‐target mutations to eliminate some genetic diseases or to express a desirable trait [176].

### 10.1 Regulatory Bodies

Regulatory bodies are constantly changing and distinct for each country and discipline. This review serves to provide a brief overview of some well‐known regulatory bodies primarily in North America at the time of writing.

In the United States, both cell and gene therapy are regulated by the United States Food and Drug Administration (FDA) Center for Veterinary Medicine in partnership with the United States Department of Agriculture (USDA) Center for Veterinary Biologics after a joint memorandum of understanding (MOU) issued in 2024. Though traditionally “biologics” are managed under the USDA, those with intended use beyond direct immune stimulation or immunotherapy for cancer are now considered to be drugs and therefore managed primarily under the FDA [177]. The overlap between jurisdictions has led to the creation of a coordinated framework for regulation in biotechnology, between the FDA, the USDA, and the environmental protection agency (EPA) in addition to the MOU [178].

One of the major concerns in cell and gene therapy regulation across nations is quality control in manufacturing processes [179]. Therefore, engagement with regulatory agents early in the process of research and development is encouraged to ensure feasibility of standardization. Approval in the US and the EU follows a risk‐based assessment. Those products that are deemed lower risk (e.g., those cell therapies that have undergone less in vitro manipulation prior to treatment) require less expansive data prior to implementation. Generally, therapies need to apply for an investigational new animal drug application to allow for research and testing before full approval prior to submitting a new animal drug application through the FDA. The USDA requires a veterinary biological product license application and to have exhibited purity, potency, safety, and efficacy testing [177].

The European Union (EU) has a similar risk‐based approach, and is regulated by the European Medicines Agency [180]. Despite a common agency, each member state of the union has different expectations for environmental risk assessments, long‐term follow‐up timeframes, vector‐specific study durations, and application processes for gene therapy [181]. This is further complicated by an evolving ecosystem with a lack of understanding of new technology and therapeutics, leading drug developers to tackle a variety of different goal posts to bring a product to an international market.

While there are many hurdles to reach the clinic floor, many agencies have purportedly attempted to encourage innovation in the area, modernizing the approach of drug and biologic approval to fit biotechnology of cell and gene therapy into the framework [182]. In one case, a streamlined FDA development process for ACTP (Animal Cells, Tissues, and Cell‐or Tissue‐Based Products) and intentional genomic alterations (IGA) has been created to facilitate decreased time to approval and implementation, known as the Veterinary Innovation Program [183]. Similar pathways are available in Japan and the EU [184, 185, 186]. However, the bar remains lower for drugs to enter the veterinary markets prior to the human market in almost all regulatory bodies, leaving an open door for the development of translational therapeutic application.

### 10.2 Performance Horse Regulations

Attempts to enhance genetic ability via gene editing is referred to as “gene doping” [187]. It is currently not legal in show horses or any equine athlete, according to all regulatory, breed, and discipline‐based organizations and has been prohibited by the International Federation of Horseracing Authorities (IFHA) and the International Federation of Equestrian Sports (FEI) [187]. This was initially broad and intended to prevent the alterations to horses' genomes to enhance metabolism, exercise physiology, disease susceptibility, or cardiovascular fitness [188]. The IFHA and FEI have both implemented an exception for in vivo or ex vivo gene therapy that is administered with the intent to treat an injury or disorder diagnosed by a veterinarian as long as it does not modify the horse's heritable genome, jeopardize welfare, or alter the horse's performance [189]. Genetic editing, such as that discussed with the implementation of CRISPR‐Cas9 systems, is banned uniformly by both organizations. Mechanisms for detecting administration of gene therapy are being developed for systemic and intra‐articular administration and involve detection of synthetic nucleic acid with HPLC‐MS, PCR, and next‐generation sequencing [99, 187, 190]. Cell therapy of unmodified cells is allowed in competition horses by the FEI, the IFHA, or the United States Equestrian Federation (USEF), as long as it is for therapeutic use [191].

## 11 Conclusion

Both cell and gene therapy show promising early indications of potential therapeutic benefits in various equine ocular diseases. However, the significant lack of controlled in vivo studies, combined with the absence of research on dose, route, and frequency, creates substantial gaps in the scientific literature. Nonetheless, with increased access to cell therapy options and the increasing presence of veterinary gene therapy (like the product SB‐001, which recently completed a clinical trial [192]), more access to data will be available imminently. Meanwhile, new therapeutics and techniques continue to develop. The approval of the first CRISPR‐Cas9 gene therapy for humans to treat sickle cell anemia and beta‐thalassemia marks the beginning of a new era in gene therapy medicine [93, 193].

The unique accessibility and immune‐privileged location of the eye, along with the high prevalence and translational qualities in equine ocular disease, create an ideal field for pursuing these novel therapeutics.

## Author Contributions

**Kimberly A. S. Young:** conceptualization, investigation, funding acquisition, writing – original draft, writing – review and editing, visualization, data curation. **Lauren V. Schnabel:** conceptualization, investigation, funding acquisition, writing – review and editing, writing – original draft, project administration, supervision. **Brian C. Gilger:** conceptualization, investigation, funding acquisition, writing – review and editing, writing – original draft, project administration, supervision.

## Disclosure

Artificial Intelligence Statement: The authors have not used AI to generate any part of the manuscript.

## Ethics Statement

The authors have adhered to the Principles of Veterinary Medical ethics as outlined by the AVMA and with the ARVO Statement for the Use of Animals in Ophthalmic and Vision Research.

## Data Availability

The authors have nothing to report.
